# Laser Induced C_60_ Cage Opening Studied by Semiclassical Dynamics Simulation

**DOI:** 10.3390/ijms12010353

**Published:** 2011-01-13

**Authors:** Hong Tang, Hongjian Li, Yusheng Dou

**Affiliations:** 1 Institute of Computational Chemistry, Chongqing University of Posts and Telecommunications, Chongqing, 400065, China; E-Mails: tanghong@cqupt.edu.cn (H.T.); lihongjian699@gmail.com (H.L.); 2 Department of Physical Sciences, Nicholls State University, PO Box 2022, Thibodaux, LA 70310, USA

**Keywords:** fullerene, laser induced fragmentation, semiclassical dynamics, cage opening

## Abstract

Laser induced opening of the C_60_ cage is studied by a semiclassical electron-radiation-ion dynamics technique. The simulation results indicate that the C_60_ cage is abruptly opened immediately after laser excitation. The opening of the C_60_ cage induces a quick increase in kinetic energy and a sharp decrease in electronic energy, suggesting that the breaking of the C_60_ cage efficiently heats up the cluster and enhances the thermal fragmentation of C_60_ fullerene.

## 1. Introduction

Fullerene (C_60_) has an icosahedral symmetry. It has a closed cage structure, which consists of 32 faces of which 20 are hexagon and 12 are pentagon. Each carbon atom in C_60_ is bonded to three others through *sp*^2^ hybridization. With this unique structure, C_60_ exhibits an extremely fast response upon laser excitation [1–3] and therefore has become a model system for studying the electronic and nuclear dynamics induced by ultrafast laser pulses [4,5].

Photoinduced fragmentation of C_60_ has attracted a great deal of interest [1–6]. Using mass spectroscopy, the fragmentation patterns of C_60_ have been well studied experimentally [2–7]. However, the mechanism behind photoinduced fragmentation is not well understood. It has been suggested that fragmentation at different laser pulse durations follows different mechanisms [7–9]. For nanosecond laser pulses, experimentally observed fragmentation patterns can be explained by statistical processes since nanosecond excitation allows the fullerene to achieve the complete equilibration of electronic energy and thermal energy through coupling between vibrational and electronic degrees of freedom [7]. For femtosecond laser pulse excitation, the excitation time scale is smaller than or similar to the electron-phonon coupling time (~250 fs) [7] and the response of the C_60_ is more complicated [8–10]. Experimental evidence shows that the relaxation following femtosecond laser excitation goes through different channels, including thermal and nonthermal fragmentations, which produce a superposition of ionized and neutral fragments [3,10,11]. It is difficult to differentiate these relaxation channels experimentally. For nanosecond laser excitations, the observed fragmentation pattern in the mass spectrum shows a series of small fragments C_n_ (*n* << 60) and a bimodal distribution of heavy fragments C_60−2_*_n_* generated by a sequential loss of a C_2_ unit [2]. For femtosecond laser pulses, a large distribution of multiple charged heavy fragments was observed and the fragmentation shows significantly different behavior [3,4]. In this communication, we report a semiclassical electron-radiation-ion dynamics (SERID) simulation study on the fragmentation of an isolated C_60_ irradiated by a 40 fs (full-width at half maximum, FWHM) laser pulse. The simulation study is focused on excitations below the continuum levels and the relaxation channels that lead to the formation of neutral fragments. Although ionization is an important channel of de-excitation, especially at high laser intensity, Jeschke and co-workers [12] concluded from phase-space argument that the processes that do not involve ionization of the C_60_ should contribute significantly to the relaxation channels if the laser intensity is not extremely high.

## 2. Methodology

In the SERID method, the state of the valence electrons is calculated by the time-dependent Schrödinger equation, but the radiation field and the motion of the nuclei are treated classically. A detailed description of this method has been published elsewhere [13–15] and only a very brief explanation is presented here. The total energy of a molecule is described by

(1) $$E_{total}=\sum_{i}^{occ}\langle \Psi_{i}|H_{0}|\Psi_{i}\rangle +\sum_{\alpha >\beta }U_{rep}(|X_{\alpha }-X_{\beta }|)$$

where the first term is electronic energy and sum goes over the occupied Kohn-Sham orbitals, which are presented by an optimized LCAO basis set. The second term is effective repulsion potential, which is approximated as a sum of two body potential as below:

(2) $$E_{rep}=\sum_{\alpha >\beta }U_{rep}(|X_{\alpha }-X_{\beta }|)$$

The Hamiltonian matrix elements, overlap matrix elements, and effective nuclear-nuclear repulsion are obtained by the density functional based tight-binding method [16]. This approach has been tested extensively for reaction energies, geometries, rotational and proton transfer barriers for a large set of small organic molecules [17] and yields very good results for homonuclear systems, like silicon and carbon, and hydrocarbon systems [18].

The one-electron states are calculated at each time step by solving the time-dependent Schrödinger equation in a nonorthogonal basis,

(3) $$i\hbar \frac{\partial \Psi_{j}}{\partial t}=S^{-1}\cdot H\cdot \Psi_{j}$$

where S is the overlap matrix for the atomic orbitals. The laser pulse is characterized by the vector potential A, which is coupled to the Hamiltonian through the time-dependent Peierls substitution [19]

(4) $$H_{ab}(X-X^{\prime })=H_{ab}^{0}(X-X^{\prime })\text{exp}\left(\frac{iq}{\hbar c}A\cdot (X-X^{\prime })\right)$$

Here *H_ab_* (X − X’) is the Hamiltonian matrix element for basis functions *a* and *b* on atoms at X and X’ respectively, and *q* = −*e* is the charge of the electron.

The nuclear motion is solved by the Ehrenfest equation of motion

(5) $$M_{l}\frac{d^{2}X_{l\alpha }}{{dt}^{2}}=-\frac{1}{2}\sum_{j}\Psi_{j}^{+}\cdot \left(\frac{\partial H}{\partial X_{l\alpha }}-i\hbar \frac{1}{2}\frac{\partial S}{\partial X_{l\alpha }}\cdot \frac{\partial }{\partial t}\right)\cdot \Psi_{j}-\partial U_{rep}/\partial X_{l\alpha }$$

where *X_l_*_α_ *=〈X̂* *_l_*_α_*〉* is the expectation value of the time-dependent Heisenberg operator for the *α* coordinate of the nucleus labeled by *l* (with *α* = *x*, *y*, *z*). Equation (5) is derived by neglecting the terms of second and higher order in the quantum fluctuations *X̂− 〈X̂* *_l_*_α_*〉*in the exact Ehrenfest theorem.

A unitary algorithm obtained from the equation for the time evolution operator [15] is used to solve the time-dependent Schrödinger equation (2). Equation (5) is numerically integrated with the velocity Verlet algorithm (which preserves phase space). A time step of 50 attoseconds was selected for this study. It was found that this time step produced energy conservation better than 1 part in 10^6^ in a one ps simulation.

The strengths of the present approach are that it retains all of the 3*N* nuclear degrees of freedom and it includes both the excitation due to a laser pulse and the subsequent de-excitation at an avoided crossing near a conical intersection. The weakness of this method is that it amounts to averaging over all the terms in the Born-Oppenheimer expansion [20–24] rather than following the time evolution of a single term. However, when the process is dominated by many electron excitations, like the interaction of the C_60_ with intense laser pulses, many electronically excited states are involved and the wave packet actually moves along a weighted-average path due to all of the electronic potential energy surfaces involved. In this case, the present approach yields very good results [25].

## 3. Results and Discussion

The initial geometry of the C_60_ was simulated for 2000 fs relaxation at 298 K using the present technique, prior to the application of the laser pulse. The calculated lengths of the double bond and single bond are 1.397 and 1.449 Å respectively, in close agreement with the experimental values [26]. The calculated HOMO-LUMO gap is 1.81 eV, which is in good agreement with the experimental value of 1.9 eV [27]. The ordering and degeneracy of the molecular orbital energy levels within 10 eV of the HOMO level are also in good agreement with experimental measurements [27]. A Gaussian shape laser pulse of 40 fs (FWHM) with a photon energy of 2.0 eV was chosen for this study. The simulation was run for an additional 1000 fs without laser to generate the initial geometries for the dynamics simulation. From this trajectory, five geometries taken at equal time intervals were selected as starting geometries. Each trajectory was propagated for 4000 fs from application of the laser pulse. Laser pulse intensity for this study is 2.55 × 10^12^ W/cm^2^. Five trajectories yielded very similar results; a representative result is presented and discussed in this paper. Bond breaking is considered to have occurred if the distance between two neighboring carbon atoms becomes greater than 1.9 Å and no recombination of these two carbons occurs thereafter. Fragmentation is deemed to have occurred if the distance between any two carbon atoms of two different fragments exceeds 1.9 Å and no subsequent bond formation between any two carbons occurs.

**Figure 1:**
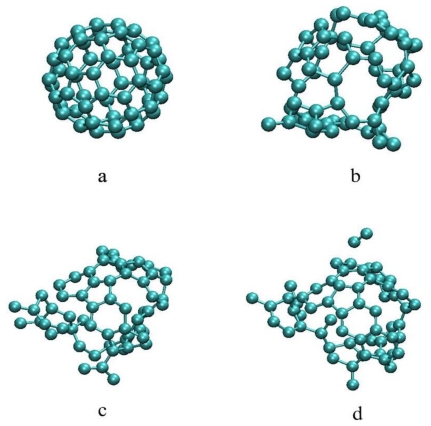
Snapshots taken from the simulation at (**a**) 0 fs, (**b**) 200 fs, (**c**) 460 fs and (**d**) 808 fs.

Four snapshots taken from the simulation at various times are shown in Figure 1. Starting from the equilibrium geometry in the electronic ground state at 0 fs, the C_60_ is electronically excited by the laser pulse. At about 200 fs (120 fs after laser irradiation), a greater number of C–C bonds have broken and the C_60_ cage has “opened up” At about 800 fs, a C_2_ dimer is observed breaking off from the C_60_ cage. Thereafter, until the end of the 2000 fs run, no further bond cleavage is observed.

**Figure 2:**
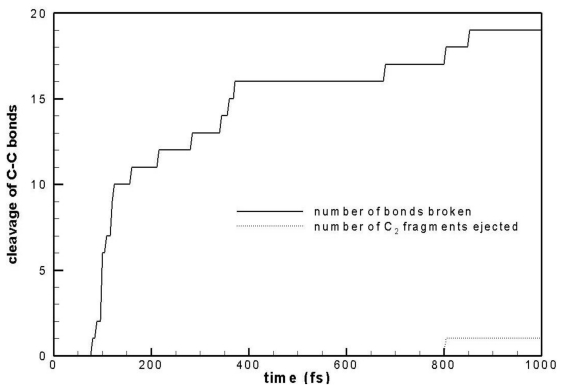
C–C bond cleavage at different times. Solid line shows the number of C–C bonds broken and dotted line shows the number of C_2_ clusters ejected.

The number of C–C bonds broken at different times is plotted in Figure 2. It is seen that extensive bond breaking occurs from 100 fs to 150 fs, immediately after laser pulse irradiation, and most bond breaking events occur before 1000 fs, including the release of a C_2_ dimer at about 800 fs. No other fragmentation is observed.

**Figure 3:**
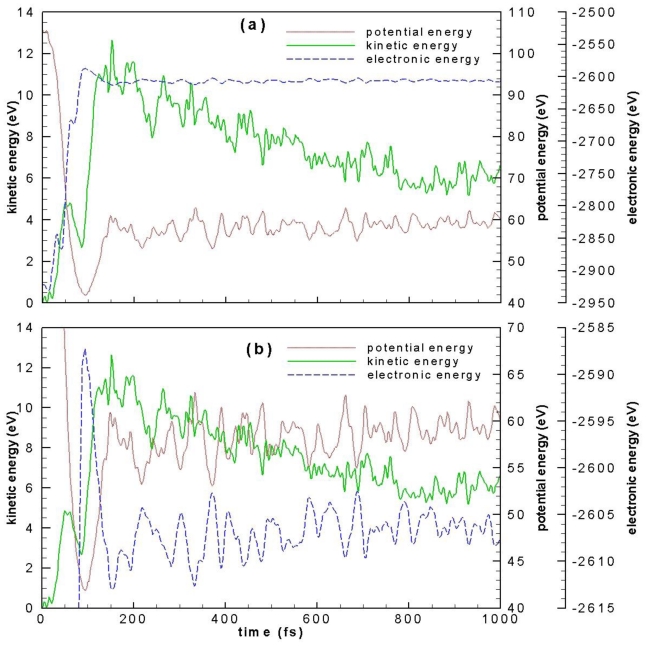
Variation with time of electronic energy, potential energy and kinetic energy. (**b**) is similar to (**a**) but shows an expanded view for electronic energy and potential energy.

Variations with time of electronic, potential, and kinetic energies are presented in Figure 3a. Figure 3b is an expanded scale for electronic energy and potential energy variations, which is compared to kinetic energy variation. Immediately after laser irradiation, electronic energy rises from about −2930 eV to −2600 eV due to the excitation of electrons from occupied molecular orbitals to unoccupied molecular orbitals while potential energy drops down 105 eV to 43 eV as a result of the expansion of the cage size. On the other hand, kinetic energy increases by about 3 eV because of the excitation of vibrational motion. It is seen from Figure 3b that from 100 fs to 150 fs there is a sharp decrease in electronic energy and a quick increase in potential energy and kinetic energy. The decrease in electronic energy must result from the extensive C–C bond breaking found in this same period of time. Electronic energy is converted to kinetic energy and potential energy through C–C bond breaking. After 200 fs, kinetic energy decreases gradually until 800 fs. This decrease is accompanied by an increase in potential energy.

The extensive bond breaking observed soon after laser pulse irradiation occurs within about 100 fs. This ultrafast process provides a decay channel for the excited C_60_. From this decay channel electronic energy is partially converted to kinetic energy. The reduction of electronic energy is due to the decrease in the energies of occupied molecular orbitals, the changes in the populations of different molecular orbitals, or both because of the breaking of chemical bonds. The increase in kinetic energy is due to the release of the energy stored in the chemical bonds broke. Consequently, the C_60_ turns out to be extremely hot, which is evidenced by the observation that kinetic energy rises up from 3 eV to 13 eV from 80 fs to 150 fs. The damaged and hot C_60_ cage may take thermal fragmentation or nonthermal fragmentation. To explain the production of the hot C_60_ cage, Laarmann and co-workers proposed that a strong shaped laser pulse triggers a multielectron excitation via the t_1g_ doorway state and the electronic excitation is followed by efficient coupling to the symmetric breathing mode of the nuclear backbone of C_60_ [28]. The simulation results presented above suggest an alternative heating mechanism: An ultrashort laser pulse induces multielectron excitation and the excited C_60_ fullerene is rapidly heated up as the C_60_ cage suddenly opens up due to the transfer of partial electronic energy into kinetic energy.

## 4. Conclusions

In summary, we performed a semiclassical electron-radiation-ion dynamics simulation study for the response of the C_60_ to ultrashort laser pulses. The simulation shows that C_60_ undergoes an abrupt opening following laser excitation. The similar behavior is also observed in the cap opening in carbon nanotubes irradiated by a femtosecond laser pulse [29,30]. The opening of the C_60_ cage leads to the conversion of electronic energy to kinetic energy and potential energy. Consequently, the C_60_ cluster is effectively heated up. These simulation results reveal a new mechanism for laser heating of the C_60_ fullerene.
